# Exploring the Interplay of the Impaction Type of the Mandibular Third Molar With Second Molar Distal Surface Caries

**DOI:** 10.1155/ijod/2749557

**Published:** 2025-06-26

**Authors:** Mohammad Amir Alizadeh Tabrizi, Amir Hossein Khazaei, Maryam Khoobyari, Mahboube Hasheminasab, Sadra Amirpour Haradasht

**Affiliations:** ^1^Department of Oral and Maxillofacial Surgery, School of Dentistry, Zahedan University of Medical Sciences, Zahedan, Iran; ^2^Oral and Dental Disease Research Center, School of Dentistry, Zahedan University of Medical Sciences, Zahedan, Iran; ^3^General Dentistry, School of Dentistry, Zahedan University of Medical Sciences, Zahedan, Iran; ^4^Department of Periodontics and Preventive Dentistry, University of Pittsburgh School of Dental Medicine, Pittsburgh, Pennsylvania, USA

**Keywords:** dental caries, impacted, molar, third, tooth

## Abstract

**Introduction:** The purpose of this study was to investigate the association of the different types of mandibular third molar impaction with the development of caries on the distal surface of the mandibular second molar.

**Materials and methods:** This prospective descriptive-analytical study was conducted among 67 participants with similar DMFT index scores referred to Zahedan Faculty of Dentistry. Third molars were categorized according to the Winter (angulation) and the Pell and Gregory (ramus relationship and impaction depth) classifications. To examine the association between the study variables, a chi-square test was performed. All statistical analyses were performed at a significance level of 5%.

**Results:** Mesioangular (35.8%), class II (67.2%), and level B (65.6%) were the most common impaction types in mandibular third molars. These impaction types were also significantly associated with the occurrence of caries on the distal surface of the mandibular second molar (*p* ≤ 0.05).

**Conclusion:** High-risk patients prone to developing distal caries in the mandibular second molar should be identified to formulate a strict screening and follow-up protocol.

## 1. Introduction

Mandibular third molar has the highest occurrence of impaction, with the prevalence rate ranging from 18% to 32% [1]. Impaction of the third molar has significant implications for oral structures. The absence of a third molar rises the likelihood of impaction in other teeth by 13 times [2]. Third molar impaction can lead to microdontia and delayed development of certain teeth [3, 4]. Cysts and tumors may develop around the impacted tooth, leading to bone destruction, while persistent inflammation in the affected area can weaken the jawbone [5]. Germectomy can be utilized in pediatric patients to prevent third molar impaction. Their results indicated reliable outcomes with insignificant postoperative complications [6].

**Figure 1:**
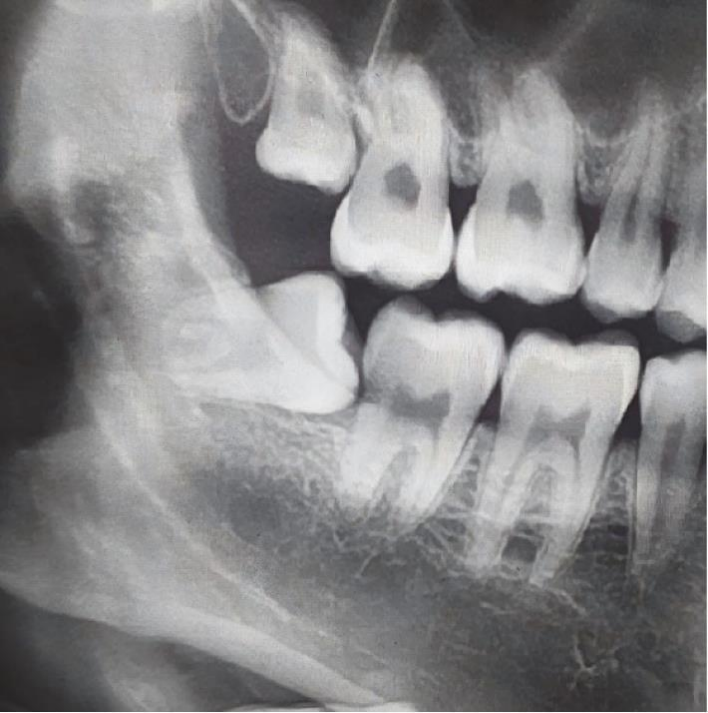
A panoramic radiograph showing a horizontal impacted wisdom tooth and dental caries affecting the distal surface of the mandibular second molar.

A complication associated with third molar impaction, as reported in various studies, is second molar distal surface caries (Figure 1) [7, 8]. When a third molar is impacted, the risk of caries increases on the distal surface of the second molar as it impinges on the cementum–enamel junction (CEJ). As a result of the challenge of visually examining the second molar distal surface when a wisdom tooth is present, diagnosing caries in this area becomes particularly difficult [7].

Mandibular third molar impaction classifications encompass the impaction depth in relation to the adjacent second molar's crown, the angle and position of the impacted tooth with respect to the second molar, and the degree to which the impacted tooth is covered by the ramus bone [9]. The purpose of this study was to investigate the association of the different types of mandibular third molar impaction with second molar distal surface caries. Additionally, this study explored the prevalence of the different types of impaction according to the Winter (angulation) and the Pell and Gregory (ramus relationship and impaction depth) classification.

## 2. Materials and Methods

This prospective descriptive-analytical research was done among individuals referred to the Zahedan Faculty of Dentistry over a 3-month period. Institutional Review Board confirmation was attained from the Zahedan University of Medical Sciences (approval number IR.ZAUMS.REC.1401.268). The current research was done in line with the Declaration of Helsinki. Written informed consent was obtained. Based on the study's objectives and using STATA software version 12, the sample size was determined to be 67, with a significance level of 0.05 and a precision level of 0.12 [10], assuming alpha = 5%, beta = 10%.

Inclusion criteria were the presence of a lower third molar, the presence of the adjacent second molar, and the absence of dental fillings in the mandibular second molar. Patients whose third molar was not classified as impacted or had cysts or tumors around the impacted third molar were excluded from further analysis. The authors also excluded the second molars with caries in all areas except the distal surface. Participants were recruited from patients aged between 20 and 45 years from all races and genders who had a similar DMFT index score (decayed, missing, and filled teeth). Since all patients were from the same city, the water fluoridation status was the same for all of them. Using this approach, we limited the confounding variables of age and variations in caries susceptibility. Pell and Gregory classification and angle identification were made possible by recruiting patients over 20 years of age, since at least two-thirds of the root is often formed in these patients [11].

Data was gathered by two skilled dentists through clinical and radiographic exams. In order to enhance intraexaminer reliability, they were calibrated prior to the investigation. Any disagreements were discussed, and a consensus was reached. Panoramic and periapical radiographs were utilized to determine the type of impaction, while bitewing and periapical radiographs were employed to detect caries. On the same computer at the same time of day, the radiographs were analyzed using a millimeter ruler to record the teeth's inclination and their relationships to the occlusal plane. Assessment of caries included examining whether it was confined to the enamel or extended into the dentin.

Different types of impacted mandibular third molars were assessed in this study based on the ramus relationship and impaction depth (Pell and Gregory's classification), which includes class I, II, III and class A, B, C, and by comparing the long axis angles in relation to the adjacent second molar (Winter's classification) [12]. Class I describes situations where the anterior edge of the ramus is behind the whole mesiodistal diameter of the third molar crown. Class II is characterized when half of the crown of the third molar is covered by the anterior edge of the ramus, and class III denotes complete coverage of the third molar crown by the ramus. Class A: the third molar's occlusal surface above or at the same level as the occlusal plane, class B: the occlusal surface of the third molar between the occlusal plane and the cervical portion of the third molar, and class C: occlusal surface of the third molar below the cervical portion of the second molar [13].

### 2.1. Statistical Analysis

Mean, standard deviation (SD), frequency, and percentage were used for reporting descriptive summaries. The collected data were entered into SPSS 28 for further analysis. Chi-square test was conducted to explore the relation among the study variables, and all statistical analyses were done at a significance level of 5%.

## 3. Results

Sixty-seven individuals with an impacted mandibular third molar were examined. The patients' mean age was 26.5 ± 3.4 years, with females making up the majority (59%) of the sample. Mesioangular (35.8%), class II (67.2%), and level B (65.6%) were the most prevalent types of impactions. Caries confined to the enamel and affecting both dentin and enamel were reported in 22.4% and 37.3% of participants, respectively.

A significant association was observed between angulation of the lower third molar and second molar distal surface caries (Table 1), with mesioangular (28.3%) and horizontal impactions (22.4%) having a greater incidence of second molar caries. Furthermore, the ramus relationship of the third molar was significantly correlated with caries on the distal surface of the second molar, with class II having a higher prevalence of second molar caries (47.8%) (Table 2). The same relationship was also observed for the third molar impaction depth, with level B having a higher prevalence of second molar caries (46.2%) (Table 3).

## 4. Discussion

The study's findings revealed that the most prevalent types of impactions, according to Winter's classification, were mesioangular position (35.8%) and horizontal position (29.9%). On the other hand, distoangular position was the least frequent type of impaction. Similar results were observed in other studies [14–16]. Regarding the Pell and Gregory classification (ramus relationship), the present study showed that class II is the most prevalent form of impaction. In contrast to the findings of this investigation, Oderinu et al. [17] reported a higher proportion of class I impaction (52.1%).

Results indicated that 22.4% of the patients had caries confined to the enamel surface, 37.3% displayed caries extending to both enamel and dentin, and 40.3% exhibited no distal caries of the second molar. Saravi et al. [18] reported a caries rate of 46.3% in the second molar when the adjacent second molar was impacted. Other studies, such as those done by Kunwar et al. [8] and Toedtling et al. [14], found that 31.8% and 38% of individuals with impacted teeth had caries in the immediate vicinity of the impacted teeth. Fallahi et al. [19] performed a similar research that reported a caries prevalence of 52% in the second molar, which was consistent with the present findings. This suggests that since brushing and maintaining good oral hygiene are more difficult in the posterior sextant of the mouth, the impaction of one tooth in this area may significantly increase the likelihood of caries in the adjacent teeth. Furthermore, due to esthetics, people tend to care less about their posterior teeth than their anterior teeth [20].

Caries prevalence was significantly higher in mesioangular and horizontal impactions. The angulation of the impacted third molar is generally thought to play a decisive role in whether tooth decay develops in the neighboring second molar. Several studies have suggested a significant association between mesioangular positioning of the mandibular third molar and increased caries rates in the second molar; more specifically, when the angle exceeds 30° (ranging from 40% to 80%), which is in line with the present study [7, 15, 17, 18, 21, 22]. It appears that more food debris and plaque can accumulate in the interproximal surface of horizontal and mesioangular impactions [23]. Second molar distal surface caries was least common in distoangular third molars in this study. This result suggests that the second molar caries may be significantly associated with the proximity of the occlusal surface of the third molar to the distal surface of second molar. Further research should examine this association. The vertical impactions are the most appropriate angulation for maintaining excellent oral hygiene [24]. In our study, a statistically significant correlation was found between the impaction type according to the Pell and Gregory classification (ramus relationship and impaction depth) and second molar distal surface caries. Caries prevalence of the second molar was significantly higher in class II and level B impactions. This is consistent with the observations of Alsaegh et al. [21] and Fallahi et al. [19]. However, in a study by Glória et al., it was concluded that level A impactions have a higher prevalence of second molar caries [25].

A limitation of this study is that the patients studied were limited to those at Zahedan Faculty of Dentistry, which may limit the generalizability of the results to other populations. In addition, a larger sample size is recommended for future studies. Further analysis could be performed utilizing the split-mouth design to investigate the association of different types of impaction with caries on the distal surface of the second molar.

## 5. Conclusion

We discovered that the ramus relationship and impaction depth (Pell and Gregory classification) and the angulation (Winter's classification) of the third molar impaction are helpful disease markers that can be used to predict the possibility of second molar distal surface caries. Thus, it is important to identify high-risk people who are prone to second molar caries in order to set up a stringent screening and monitoring procedure.

## Figures and Tables

**Table 1:** Association between the angulation of mandibular third molar and caries on the distal surface of mandibular second molar.

Caries	Yes		No	*p*-Value
Angulation	Enamel *n* (%)	Enamel and dentin *n* (%)	*n* (%)	
Mesioangular	8 (11.9)	11 (16.4)	5 (7.5)	0.003*⁣*^*∗*^
Distoangular	0 (0)	1 (1.5)	3 (4.5)	
Vertical	2 (3.0)	3 (4.5)	15 (22.4)	
Horizontal	5 (7.5)	10 (14.9)	4 (6.0)	
Total	15 (22.4)	25 (37.3)	27 (40.3)	

*⁣* ^*∗*^Significant results (*p* ≤ 0.05).

**Table 2:** Association between the ramus relationship of the mandibular third molar and caries on the distal surface of the mandibular second molar.

Caries	Yes		No	*p*-Value
Ramus relationship	Enamel *n* (%)	Enamel and dentin *n* (%)	*n* (%)	
I	3 (4.5)	4 (6.0)	14 (20.9)	0.018*⁣*^*∗*^
II	12 (17.9)	20 (29.9)	13 (19.4)	
III	0 (0)	1 (1.5)	0 (0)	
Total	15 (22.4)	25 (37.3)	27 (40.3)	

*⁣* ^*∗*^Significant results (*p* ≤ 0.05).

**Table 3:** Association between the impaction depth of the mandibular third molar and caries on the distal surface of the mandibular second molar.

Caries	Yes		No	*p*-Value
Impaction depth	Enamel *n* (%)	Enamel and dentin *n* (%)	*n* (%)	
A	3 (4.4)	6 (8.9)	9 (13.4)	0.007*⁣*^*∗*^
B	13 (19.4)	18 (26.8)	13 (19.4)	
C	0 (0)	1 (1.5)	5 (7.5)	
Total	16 (23.8)	25 (37.3)	27 (40.3)	

*⁣* ^*∗*^Significant results (*p* ≤ 0.05).

## Data Availability

The research data can be provided upon request.
